# Dermatofibrosarcoma protuberans: unmasking a rare clinical image

**DOI:** 10.11604/pamj.2024.48.112.44074

**Published:** 2024-07-17

**Authors:** Shivali Kalode, Prerna Tekulwar

**Affiliations:** 1 Department of Pathology, Jawaharlal Nehru Medical College, Datta Meghe Institute of Higher Education and Research, Sawangi (Meghe), Wardha, Maharashtra, India

**Keywords:** Dermatofibrosarcoma protuberans, spindle cells, storiform pattern

## Image in medicine

Dermatofibrosarcoma protuberans (DFSP) is a rare, slow-growing, soft tissue tumor, with a prevalence of 0.8-4.5 cases per 1 million per year. As the name suggests, the tumor often involves the dermis and soft tissues, with pedunculation and spread in advanced stages. It accounts for between 1% and 6% of all soft tissue sarcomas and 18% of all cutaneous soft tissue sarcomas. Here, we report a case of a 72-year-old male, who presented in the dermatology outpatient department with an exophytic overgrowth over his right elbow in the last 15 years which has progressively increased in size with a noncontributory family history. Physical examination revealed a lobulated, soft, non-tender, and mobile mass over the right elbow. Surgical excision was performed under local anesthesia. The excised specimen measured 6 x 5.5 x 1.5 cm. The specimen was sent to the histopathology section of the Department of Pathology. Microscopically, H&E staining revealed predominantly spindle cells arranged in storiform pattern, cytoplasm is moderate and eosinophilic with oval to elongated, hyperchromatic nuclei. Intervening fibrous septa and inflammatory infiltrate are also seen. Since metastatic potential is common, follow-up every 6 months was advised.

**Figure 1:**
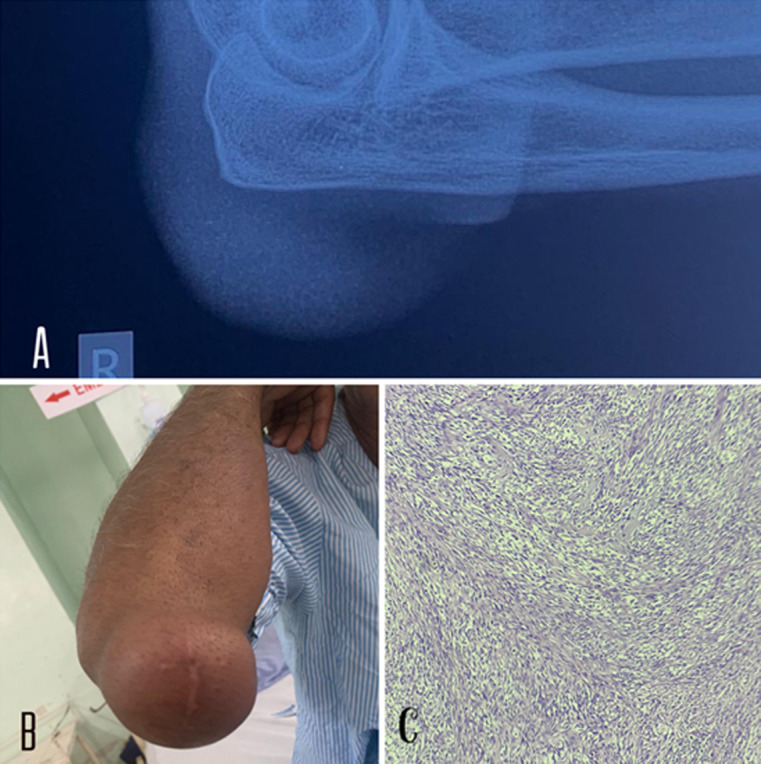
A) radiological image; X-ray showing soft tissue swelling noted around the elbow joint; with no evidence of fracture or bone abnormalities noted; B) clinical image showing lobulated, soft, non-tender, and mobile mass over the right elbow; C) microscopic low power view (10X) image of an excised specimen of mass over right elbow; H&E staining revealed predominantly spindle cells arranged in a storiform pattern with moderate eosinophilic cytoplasm and hyperchromatic oval to elongated nuclei

